# Exercise prior to a freely requested meal modifies pre and postprandial glucose profile, substrate oxidation and sympathovagal balance

**DOI:** 10.1186/1743-7075-8-66

**Published:** 2011-09-24

**Authors:** Keyne Charlot, Aurélien Pichon, Didier Chapelot

**Affiliations:** 1 Université Paris 13, Laboratoire des Réponses Cellulaires et Fonctionnelles à l'Hypoxie, UFR SMBH, 74 rue Marcel Cachin, 93017, Bobigny, France

**Keywords:** Exercise, preprandial glucose decline, interstitial glucose, postprandial glucose, fat oxidation, heart rate variability, sympathovagal balance, freely requested meal

## Abstract

**Background:**

The effects of exercise on glucose and metabolic events preceding and following a freely initiated meal have never been assessed. Moreover, the relationship between these events and sympathovagal balance is not known. The objective of this study was to determine whether exercise prior to a freely requested meal modifies the pre- and postprandial glucose profile, substrate oxidation and sympathovagal balance.

**Methods:**

Nine young active male subjects consumed a standard breakfast (2298 ± 357 kJ). After 120 min, they either performed 75 min of exercise on a cycle ergometer (EX - 70% VO_2max_) or rested (RT). Lunch was freely requested but eaten *ad libitum *only during the 1^st ^session, and then energy intake was fixed across conditions. Glucose and sympathovagal balance were assessed continuously using a subcutaneous glucose monitoring system and analysis of heart rate variability, respectively. Every 5 min, a mean value was calculated for both glucose and sympathovagal balance. Substrate oxidation was determined by calculating the gas exchange ratio when lunch was requested and 180 min after the onset of eating.

**Results:**

Preprandial glucose profiles were found in 72% of the sessions and with a similar frequency under both conditions. Meals were requested after a similar delay (40 ± 12 and 54 ± 10 min in EX and RT respectively; ns). At meal request, sympathovagal balance was not different between conditions but CHO oxidation was lower and fat oxidation higher in EX than in RT (-46% and +63%, respectively; both p < 0.05). Glucose responses to the meal were higher in incremental (+ 48%) but not in absolute value in EX than in RT, with a higher fat oxidation (+ 46%, p < 0.05), and a greater vagal withdrawal (+ 15%, p < 0.05).

**Conclusions:**

These results show that exercise does not impair preprandial glucose declines at the following meal freely requested, but leads to an increased postprandial glucose response and an elevated fat oxidation, an effect that vagal withdrawal may contribute to explain.

## Background

Exercise is now considered to contribute to both the reduction in the prevalence of type 2 diabetes and the improvement of glucose tolerance [1]. The effect on postprandial glucose was observed even after a single bout of exercise [2,3]; however, the delay between the exercise session and the test meal is an important parameter. In the immediate post-exercise period and up to 90 min later, postprandial glucose concentrations have been reported to be increased [4-11] or unchanged [5,12-17]. This is thought to be mainly the consequence of reduced insulin concentrations [5,7,13,14]. Another hypothesis is that exercise may transiently blunt glucose tolerance by changing the sympathovagal balance. Prior exercise has been shown to stimulate postprandial sympathetic activity [18], leading to a reduction in pancreatic insulin release [19]. In addition, partially impairing vagal activity before a meal resulted in reduced glucose tolerance [11,20], indicating that an exercise-induced withdrawal of vagal activity may also contribute to this effect either directly or indirectly by alleviating the inhibition of sympathetic stimulation [21]. The continuous evaluation of sympathovagal balance is now possible with the analysis of heart rate variability (HRV), a non-invasive method that investigates the autonomic modulation *via *changes in RR intervals [22] and may be employed to gain insight into the mechanism underlying exercise-mediated glucose tolerance.

Exercise is usually not followed by an increase in hunger or energy intake [23-31]. In some studies, hunger scores were even found to be briefly reduced [23-25]. The mechanism for this absence of energy compensation is not known. Moreover, a longer delay of meal initiation after exercise has been reported [24]. Thus, it is important to study the sequence preceding meals spontaneously initiated. In animals [32] and humans [33-36], meals are requested after a decline in blood glucose, illustrating central glucopenia [37]. It was found that this phenomenon could even discriminate between meals and snacks [33]. To this day, a possible modification of the glucose profile by prior exercise has not been studied. To detect these preprandial glucose declines, a continuous blood withdrawal system allowing measurements every 5 min is required. However, this method is not fully compatible with exercise. Recently, using a subcutaneous glucose monitoring system (CGMS^®^), these preprandial glucose declines were measured under everyday life conditions [38]. This system represents a promising alternative and is already being used to monitor glucose profiles during exercise in young diabetic patients [39].

The current study was planned with two main objectives. The first was to determine whether prior exercise would alter the neurometabolic state in which a meal is spontaneously initiated. The second was to verify whether the glucose response to this meal would be altered by prior exercise and whether it was accompanied by a change in fat oxidation and sympathovagal balance.

## Methods

### Subjects

Nine healthy, moderately active men were recruited (see Table 1 for characteristics) using board advertisements in the University area. Their fat mass was measured using an 8-electrode bioelectrical impedance analyzer (Tanita BC 418MA, Tanita Co. NL), and their restrained eating habits were assessed using the Three-Factor Eating Questionnaire [40]. The number of subjects in the study was based on previous studies showing that significant differences in the delay of meal initiation after exercise were reported with 8 subjects [29,33]. The subjects were informed about the nature and risks of the experimental procedure prior to giving their written consent. The protocol was officially approved by the representatives of the local arm of the National Ethics Committee (Comité de Protection des Personnes Ile-de-France n°X)

**Table 1:** Subjects' characteristics (n = 9)

Age (yr)	21.9 ± 1.8
Body weight (kg)	73 ± 6
Height (m)	1.80 ± 0.06
Body mass index (kg.m^-2^)	22.7 ± 1.6
Body fat (%)	13.4 ± 1.5
Restrained eating score*	3.3 ± 2
VO_2max _(mL.kg^-1^.min^-1^)	49 ± 9

Values are expressed as mean ± SD.

*F1 score on the Three Factor Eating Questionnaire [40].

### Study design

**Figure 1:**
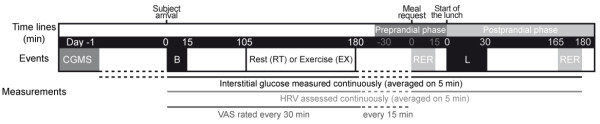
**Schedule of experimental events for each subject**. The experimental procedure was conducted on 3 successive days with rest (RT) and exercise (EX) as conditions tested in counterbalanced order across subjects (D1 and D3) and separated by a washout day (D2). CGMS, continuous glucose monitoring system; B, breakfast; VAS, visual analogue scales; HRV, heart rate variability; L, freely requested lunch; RER, respiratory exchange ratio.

The study followed a within-subject design with individuals completing the following 2 test conditions in a counterbalanced order: 75 min of exercise (EX) or 75 min of rest (RT). The 2 conditions were completed on the 1^st ^and 3^rd ^of 3 consecutive days with a washout day between them so that the glucose monitor system (CGMS^®^) was inserted only once and kept in place. The procedure is described in Figure 1. On the evening prior to the first test day, the CGMS^® ^sensor was inserted in the subject's lateral abdominal wall. On the test days (D1 and D3), the subject arrived at the laboratory unit at 08:00 after an overnight fast. He was equipped with a cardiofrequencemeter and then ate his breakfast at 08:30. At 10:15, depending on the randomization; he either rested on a chair (RT) or performed exercise (EX) for 75 min. At 11:30, the subject was placed in an isolated room without temporal cues and asked to spontaneously request his lunch when he felt the need to eat. This procedure is the classic one used in studies of spontaneous eating behavior that has been shown to be sensitive to macronutrient or pharmacological manipulations [41,42]. Immediately after the subject requested his lunch, gas exchange was measured for 15 min prior to lunch being served. The time for eating this meal was limited to 30 min. A second 15 min gas exchange measurement was planned for the 165^th ^min following the onset of lunch. The cardiofrequencemeter was then removed, and the subject was allowed to leave the laboratory. On the 3^rd ^day (D3) of the study, a similar session took place with the second condition. The CGMS^® ^was removed on D3 after the final gas exchange measurement.

### Preliminary testing

At least 3 days prior to the experimental trial, individuals' maximal oxygen consumption (VO_2max_) was determined using a maximal workload test conducted on a bicycle ergometer (Ergoselect 100P, Ergoline, Bitz, Deutschland). Subjects wore a facemask (Hans Rudolph, 8940 Series, Kansas City, KA, USA), and gas exchange rates were measured using an open-circuit spirometry V_max _Encore (Viasys Healthcare, Palm Springs, CA, USA). The same equipment was used for all calorimetry measurements throughout the study. The VO_2max _was considered to be reached when two of the following criteria were met: 1) a less than 2 mL·kg^-1^·min^-1 ^VO_2 _increase despite workload increase; and 2) a respiratory energy ratio (RER) > 1.15.

### Foods

On the eve of each test day, subjects were required to eat their dinner at home at the same hour. The 2 meals consisted of traditional main dishes (couscous and paella) with similar carbohydrate (CHO), fat and protein compositions (33, 43 and 24 energy percent [En%], respectively). Beverages, dessert and bread were freely added by the subjects before the first session; however, participants were instructed to keep their dinner meals similar before the next session.

On the day of the test, the energy content of the breakfast meal was calculated based on each participant's usual intake at this meal, which ranged from 1700 to 2900 kJ. The same foods were provided for each subject (i.e., bread, butter, marmalade, fruit yogurt and sweetened milk) equal to the energy value calculated for each subject. In addition, the macronutrient composition (in %) was matched but with an energy value corresponding to their usual breakfast.

The lunch meal consisted of ground beef mixed with mashed potatoes ("hachis parmentier"), bread and fruit yogurt. Subjects were allowed to eat *ad libitum *on the first day but were required to eat exactly the same amount of each item and at a similar rate on the second test day. The mean energy intake at both breakfast and lunch are shown in Table 2. The 2 conditions were counterbalanced, and the amount served at lunch during the second session was determined after EX for 4 subjects and after RT for 5 subjects. While this may have led to a higher or lower energy load compared to the one that would have been spontaneously consumed, this method was considered to respect the physiological state better than a fixed amount or an amount based on individual body weight and a putative fixed daily energy distribution.

**Table 2:** Energy and macronutrient intake at each experimental meal

	Breakfast	Lunch
Energy (kJ)	2298 ± 357	5225 ± 998
CHO (g)	92.8 ± 15.6	117.4 ± 22.1
CHO (%)	67.4 ± 1.7	37.8 ± 3.9
Fat (g)	12.7 ± 1.8	63.9 ± 14.1
Fat (%)	20.9 ± 1.6	45.8 ± 3.5
Protein (g)	16.0 ± 2.4	51.3 ± 10.2
Protein (%)	11.7 ± 0.4	16.4 ± 0.4
% of TDEE	22 ± 4	49 ± 11

Values are expressed as mean ± SD. The experimental meal was *ad libitum *on the first session, but consumed in the same amount than in the first session on the second session. CHO, carbohydrate. % of TDEE, percentage of total daily energy expenditure calculated using the Harris & Benedict equation for resting metabolic rate (RMR) corrected for moderate activities (RMR × 1.4).

### Exercise

After a 5 min warm-up period at 75 W, the workload was progressively increased for a 10 min period until the subject reached 70% of his VO_2max_. This intensity was then maintained for 60 min. Continuous gas exchange allowed for the measurement of energy expenditure (EE) and constant adjustment of the workload so that exercise was maintained at the desired intensity.

### Motivation to eat

From the beginning of breakfast to the end of the 75 min of exercise or rest, motivation to eat was assessed on 100 mm visual analogue scales (VAS) addressing the questions ''Do you feel hungry?'' (hunger scale), ''Do you want to eat something?'' (desire to eat scale) and ''How full do you feel?'' (gastric fullness scale) every 30 min for 3 hours, and then every 15 min until a meal was requested. These scales were anchored with "not at all" and "extremely" at the left and right ends, respectively. The distance between the extreme left and the subject's vertical dash represented the rating score, expressed in mm.

### Glucose measurement

A continuous glucose monitoring system (CGMS^®^, Medtronic Minneapolis, USA) was used to determine the glucose profile. It consists of an electrochemical sensor with glucose oxidase immobilized on an electrode. Interstitial glucose is measured every 5 min. Four calibrations on venous blood taken from the fingertip during a stable state period (i.e., not in the postprandial state) were carried out every 24 h using the glucometer, Optium Xceed (Witney, Oxon, UK). Interstitial glucose has been reported to be a valid surrogate for the blood glucose level [43] and allows transient glucose fluctuations to be assessed. It is important to note that a lag, varying from 4 to 10 min, between plasma and interstitial glucose levels has been reported, with the former usually preceding the latter [44]. The duration of the lag seems to depend on the glucose level and kinetics. The lag between 2 CGMS^® ^sensors is also important. However, no fixed value has been proposed to this day. Thus, we decided to present and analyze the glucose data without an arbitrary lag.

### Energy expenditure and substrate oxidation

At rest, EE was calculated using the energy equivalent of O_2 _derived from the Weir equation [45] and substrate oxidation was calculated using the Péronnet & Massicotte equations [46] with the assumption that protein oxidation is negligible. During exercise, the Jeukendrup & Wallis equation [47] for moderate to high-intensity exercise (50-75% VO_2max_) was used to calculate CHO oxidation and EE. This stoichiometric equation is more appropriate to exercise since it takes into account that only 20% of the glucose oxidized is derived from plasma, with 80% being provided by glycogen. Allowing a delay of 10 min to reach stability, only the last 5 min of the preprandial and the postprandial measurements were used for analyses.

### Assessment of the autonomic nervous system

Autonomous modulation was evaluated by the frequency domain analysis of HRV. The RR intervals were recorded during the day using a cardiofrequencemeter (T6, Suunto, Finland), stored for analysis and then screened for artifact (less than 2%). The determination of a suitable series of 256 RR intervals for each 5 min generated indices of HRV that correspond with each value measured by the CGMS^®^. Power spectral analyses were performed with the HRV Analysis Software 1.1 for Windows [48]. Total power in the frequency range (0 - 0.40 Hz) was divided into low frequency (LF: 0.04 - 0.15 Hz) and high frequency (HF: 0.15 - 0.40 Hz) bands. The LF band has been attributed to both the vagal and sympathetic modulations, the HF band to vagal modulation and the LF to HF ratio (LF/HF) to the sympathetic modulation of total activity [49]. The use of normalized units (nu) for the HF component (HFnu = (HFms^2^/(LFms^2 ^+ HFms^2^) × 100) has been recommended [22]. Our subjects breathed spontaneously but reproducibility has been shown to be similar between spontaneous and paced breathing techniques [50].

### Data analysis

Preprandial glucose declines (PPGD) were based on the definitions of Melanson et al. [34,35]: at least 5% magnitude and 5 min duration. Since the CGMS^® ^only provided an average value every 5 min, and in accordance with our observations from a previous study [33], we decided that the criteria needed to be more conservative. Therefore, we decided that PPGD should be defined by a decline that lasted at least 10 min (from 2 consecutive time points) and that the meal had to be requested before the return of glucose concentrations to the basal level.

Areas under the curve (AUC) were calculated by the trapezoidal method over the 180 min following the start of the lunch meal for both the glucose and HRV indices. For glucose, two postprandial AUCs were calculated: an incremental area (values minus basal level, AUC_inc_), and an absolute level area (AUC_abs_). The first was used to specifically determine the glucose response to the meal and the second to evaluate whether differences in this response actually resulted in differences in glucose levels.

### Statistics

All variables means and AUCs were compared using paired Student's *t-*tests. Glucose and HRV indices profiles were analyzed using analyses of variance (ANOVA) for repeated measures with condition (RT and EX) and time as within-subject factors. According to the proposed approach of analysis of serial measures [51], time was divided into 4 periods of interest: 1) from breakfast to rest or exercise, 2) rest or exercise and the delay until one subject asked for his meal (the interruption of the comparison is due to the reduction in the sample size), 3) prelunch and 4) postlunch. The prelunch period consisted of the 45 min preceding meal intake (i.e., 30 min before the lunch request and 15 min during gas exchange measurement). The postprandial period was divided into 30 min periods (6 time points). When an effect was significant, appropriate comparisons using Scheffe's tests were conducted. Statistical significance was set at p < 0.05. All results are expressed as mean ± SEM, unless otherwise stated. All data were obtained for 9 subjects except postprandial HRV during which the recording failed for 1 subject.

## Results

### HRV indices, energy expenditure and substrate oxidation during the rest or exercise period

Values and statistical significances are displayed in Table 3. Compared to rest, exercise induced an increase in energy expenditure of 2690 ± 226 kJ with 136 ± 12 g CHO and 11 ± 2 g fat oxidized. During exercise, LF and HF decreased but the LF/HF ratio increased, demonstrating the shift from sympathovagal balance toward sympathetic activation. As expected, HR dramatically increased during exercise.

**Table 3:** HRV indices, energy expenditure and substrate oxidation during the rest or exercise period

	Rest	Exercise
HR (bpm)	64 ± 4	151 ± 3‡
LF (ms^2^)	2443 ± 184	68 ± 20‡
HF (ms^2^)	2952 ± 757	41 ± 18†
HF (n.u.)	47 ± 6	21 ± 3†
LF/HF	1.7 ± 0.5	8.2 ± 1.8*
Energy (kJ)	418 ± 39	3109 ± 222‡
RER	0.807 ± 0.008	0.931 ± 0.003‡
CHO (g)	10.7 ± 3.4	146.3 ± 11.2‡
Fat (g)	6.3 ± 0.6	17.6 ± 1.4*

Values are expressed as mean ± SEM. HR, Heart rate, LF, Low-frequencies. HF, High-frequencies. RER, respiratory exchange ratio. CHO, carbohydrates. Significantly different from RT, * p < 0.05, † p < 0.001, ‡ p < 0.01

### Appetite, meal request delay and preprandial glucose declines

**Figure 2:**
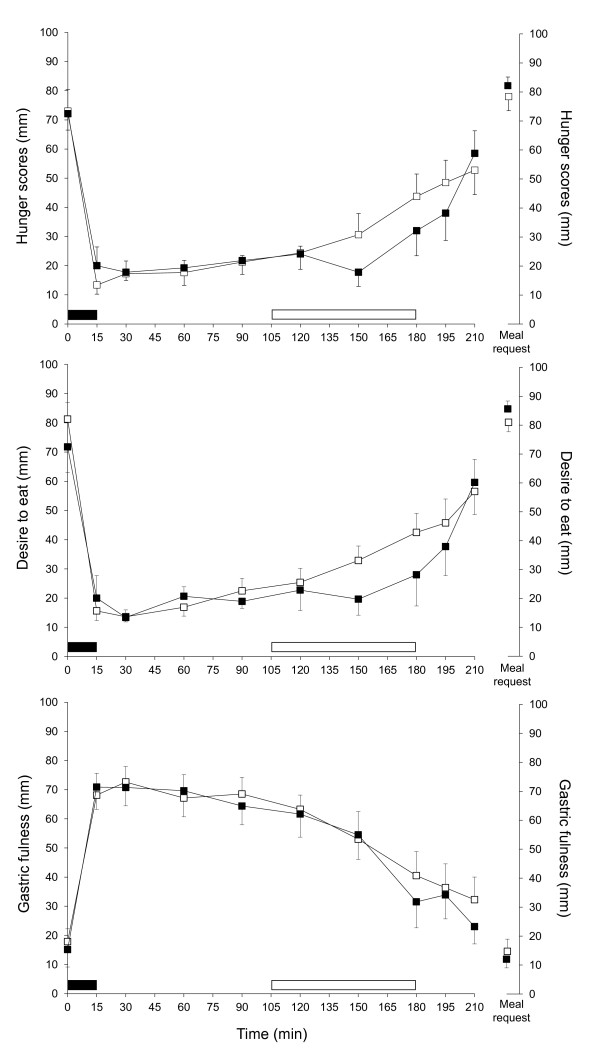
**Motivation to eat from the breakfast to the meal request (in mm)**. Hunger (A), desire to eat (B) and gastric fullness (C) scores on each visual analogue scale. The profile was stopped when one subject requested his meal. The black rectangle indicates breakfast intake periods and white rectangle indicates the 75 min of exercise or rest. All results are mean ± SEM.

**Figure 3:**
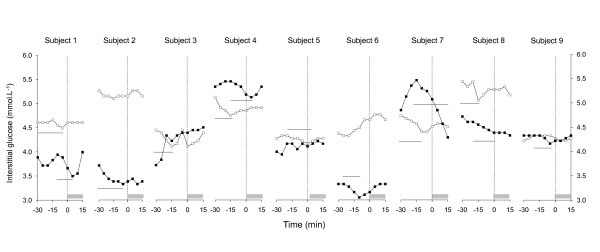
**Individual preprandial interstitial glucose concentrations**. T = 0 min indicates the meal request. The grey rectangles indicate the 15 min gas exchange periods. The lines indicate the profiles that fulfilled the Melanson et al. criteria for preprandial glucose declines [34] modified for the present study.

The delay from the end of the exercise or rest period until the lunch request was not significantly different between conditions (40 ± 12 and 54 ± 10 min for RT and EX, respectively; ns). Hunger, desire to eat and gastric fullness scores are illustrated in Figure 2. The ANOVA revealed no effect of condition. Based on previous results and on the differences observed in the mean values of our data, comparisons were conducted between conditions at 150, 180 and 195 min. However, they failed to reach significance. Preprandial glucose profiles are shown in Figure 3. According to the defined criteria, 13 of the 18 lunch requests were preceded by a PPGD. The mean magnitude of the trough was 8 ± 1% and the mean delay between the onset of the PPGD and the meal request was 25 ± 3 min. Four of the subjects had a PPGD in both conditions, 3 only in EX and 2 only in RT. In 3 of the 5 profiles without PPGD, a glucose decline was observed but lunch was requested after the value had returned to the baseline level. In 2 of the profiles, the magnitude and duration were less than the required values.

### Interstitial glucose

**Figure 4:**
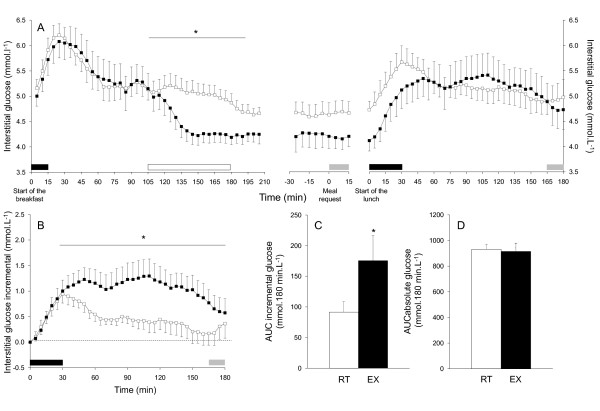
**Mean interstitial glucose profiles**. The results are displayed in absolute values from the breakfast to 180 min after the lunch (A) and during the postlunch period only for the incremental values (B). Areas under curves (AUC) are presented in incremental (C) and absolute (D) values during the postlunch period. The black rectangles indicate breakfast and lunch intake periods, the white one indicates the 75 min of exercise or rest and the grey ones indicate the 15 min gas exchange periods. All results are mean ± SEM. *EX significantly different from RT, p < 0.05.

Glucose profiles are shown in Figure 4A for the absolute values from the beginning of the breakfast to the end of the experimental session (3 h after the lunch) and in Figure 4B for the incremental values during the postlunch period. Analyses of the glucose profiles showed a significant interaction between condition and time (p < 0.001). Comparisons indicated that the interstitial glucose began to decrease when the exercise intensity reached 70% VO_2max _(120 min) and was lower in EX than in RT until the end of the exercise (all p < 0.05). This difference was still significant 25 min after the end of the exercise period but failed to reach significance in the preprandial period. During the postlunch period, the first glucose peak was reached later in EX than in RT (67 ± 9 and 33 ± 4 min, respectively; p < 0.005). Moreover, its magnitude was higher in EX than in RT when calculated in incremental units (1.62 ± 0.30 and1.09 ± 0.16 mmol·L^-1^, respectively; p < 0.05), but this difference was not significant in absolute values (5.83 ± 0.29 and 5.74 ± 0.43 mmol·L^-1^, respectively; ns). Analyses of the postprandial glucose profiles showed a significant interaction between condition and time (p <0.001). Comparisons indicated that the glucose response to the meal (i.e., the incremental profile) was higher in EX than in RT between 30 and 180 min (all p < 0.05). Consistently, the glucose incremental AUC (Figure 4C) in EX was higher than in RT (p <0.05). No effect of condition was found on the glucose profiles for absolute values and no difference in the absolute AUC (Figure 4D) was observed between conditions.

### Pre and postprandial energy expenditure and substrate oxidation

Results are presented in Table 4. At meal request, RER and CHO oxidation were lower by 10% and 46%, respectively; and fat oxidation was higher by 63% (all p < 0.05) in EX than in RT. Three hours after lunch, RER and CHO oxidation were still lower by 8% and 37%, respectively; and fat oxidation was higher by 46% (all p <0.05) in EX than in RT.

**Table 4:** Energy expenditure and substrate oxidation at meal request and 3 h after the lunch meal

	At meal request		3 h after lunch	
	**Rest**	**Exercise**	**Rest**	**Exercise**

EE (kJ/min)	5.96 ± 0.12	5.96 ± 0.31	6.93 ± 0.17	6.71 ± 0.23
RER	0.870 ± 0.021	0.784 ± 0.027*	0.885 ± 0.027	0.818 ± 0.030*
CHO (g/min)	0.22 ± 0.03	0.12 ± 0.04*	0.28 ± 0.05	0.18 ± 0.04*
Fat (g/min)	0.06 ± 0.01	0.10 ± 0.02*	0.07 ± 0.01	0.10 ± 0.02*

Values are expressed as mean ± SEM. EE, Energy expenditure. RER, respiratory exchange ratio. CHO, carbohydrates. Significantly different from RT, * p < 0.05

### Pre and postprandial heart rate variability indices

At meal request, an effect of condition was found only for HR (p < 0.005), and an interaction between time and condition for LFms^2 ^(p < 0.05). Comparisons showed that LF was higher in RT than in EX until 15 min prior to the lunch request (p < 0.05) but this difference was not observed afterwards (data not shown). HR was higher in EX than in RT from 30 min prior to the meal request until 15 min after the meal (p < 0.05, data not shown).

**Figure 5:**
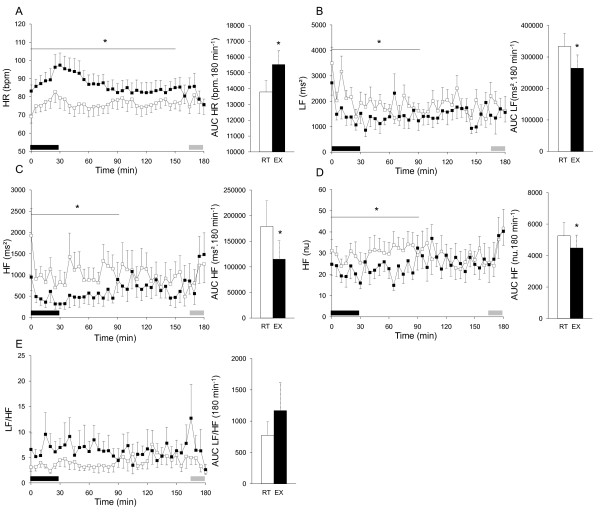
**Time courses and area under curves of postlunch heart rate variability (HRV) indices**. Heart rate (A, HR, in bpm), low-frequency (B, LF, in ms^2^), high-frequency (HF) in ms^2 ^(C) and in normalised units (D, HFnu), and LF/HF ratio (E). The black rectangles indicate lunch intake period and the grey rectangles indicate the 15 min gas exchange periods. All results are mean ± SEM. *EX significantly different from RT, p < 0.05.

After lunch, a rapid increase in HR and a decrease in HFms^2^, HFnu and LFms^2 ^were observed. The ANOVA showed an effect of condition for HR (p < 0.001), LFms^2 ^(p < 0.01), HFms^2 ^(p < 0.05) and HFnu (p < 0.05). An interaction between condition and time was found for HR (p < 0.005) and HFms^2 ^(p < 0.05). Comparisons between the postprandial HRV profiles for 30 min periods showed that HR was higher in EX than in RT during the first 150 min and that HFms^2^, LFms^2 ^and HFnu were lower in EX than in RT during the first 90 min of the postprandial interval (all p < 0.05, Figures 5A, B, C, D and E). When AUCs were analyzed, comparisons showed that postprandial HRV indices in EX were higher than in RT for HR (by 12%, p < 0.001) and lower for HFms^2^, HFnu (by 46% and 15%, respectively; both p < 0.05) and LFms^2 ^(by 21%; p < 0.001).

## Discussion

In this study, sympathovagal modulation and interstitial glucose concentrations were for the first time recorded continuously and in parallel to assess the effects of exercise on the pre- and postprandial sequences of a spontaneously requested meal. Our results show, firstly, that in young male adults, exercise did not alter the preprandial sequence (delay, motivation to eat, preprandial glucose decline) but that under this condition the meal was requested in a metabolic state that was characterized by a higher proportion of energy being derived from fat. Secondly, the postprandial glucose response was increased in relative but not absolute values after exercise, and this was associated with an increase in fat oxidation and vagal withdrawal.

### Prelunch results

Exercise did not alter the motivation to eat or delay request for the meal. After a similar physical workload, exercise has been reported to reduce hunger ratings in some [23-25] but not all studies [26-31]. This effect was brief (<10 min) and no longer present at meal onset. Although these results were reported for a study with a similar number of subjects, the non-significant trend that we observed in the immediate post-exercise period for hunger and desire to eat suggests that a larger sample size may be required. While a slightly longer delay (5 min) until eating onset has been previously reported [24], this delay was not significantly changed in our study. Again, a higher number of subjects may be required in order to observe this effect. Therefore, specific research into this matter is needed in the future.

One of our hypotheses was that the glucose decline preceding a meal request would be altered by prior exercise. When an exercise that depleted muscle glycogen was performed on the day before testing, it has been reported that most meals were requested without a prior glucose decline [35]. This was accompanied by a very low RER that was corrected after re-feeding. According to the criteria proposed by Melanson et al. [34,35] that we adapted to be more conservative, a glucose decline was observed before 72% of lunch requests. In the other cases, glucose actually decreased during the preprandial interval, but the magnitude and duration of our modified criteria were not fulfilled. The mean delay between the onset of this preprandial glucose decline and the meal request was consistent with previous observations [33-35]. Exercise did not seem to impair this preprandial glucose profile since it was observed with a similar frequency in both conditions. However, our exercise was not designed to deplete muscle glycogen. Based on our glucose oxidation results (~ 146 g) and the energy partitioning proposed by Jeukendrup [47] (i.e., 80% of the glucose oxidized derived from glycogen) and the glucose oxidized at rest (~ 11 g), ~ 106 g of glycogen was used [(146 × 0.8) - 11]. According to the subjects' leg muscle mass, which was estimated at ~ 21 kg by the bioelectrical impedance analyzer, and a mean glycogen muscle concentration of 150 mmol/kg wet weight, it can be assumed that the exercise depleted glycogen of the whole leg muscle mass by only 18%. In our study, the RER also decreased after exercise when compared with the rest condition, but this did not prevent the occurrence of glucose declines. Therefore, it seems that the glycogen status, more than the exercise *per se*, is the reason behind the absence of a preprandial glucose decline, or more likely, the absence of its detection.

It must be noted that there was a much larger glucose decline at the onset of the exercise session (from ~ 5.0 to ~ 4.2 mmol.L^-1^), a well-known phenomenon mediated by the increase in muscle glucose uptake [52]. This occurs despite the fact that liver potently increases its glucose output due to lower insulin and higher glucagon and catecholamine secretion [52]. However, when preprandial glucose declines were observed, glucose concentrations were in a stable state in each subject.

Between the end of exercise and the meal request, carbohydrate oxidation was 46% lower than in the rest condition. Considering that ~ 20% of the CHO oxidized during exercise came from blood glucose [47], ~ 20 g of glucose needed to be compensated for, compared to the rest condition. Although the post-exercise reduction in glucose oxidation could not completely account for the compensation of this glucose difference, it may contribute to preclude an earlier preprandial glucose decline and meal request.

Glucose [36] and ghrelin [53] are the main putative determinants of meal initiation. We have previously reported that ghrelin is increased before meal request [54] but there are arguments against its role as a necessary factor in meal initiation [55]. Moreover, ghrelin was not found to be altered after an exercise session of intensity and duration similar to the one used in our study [56]. Cholecystokinin and glucagon-like peptide-1 have been reported to be increased after a single bout of exercise [11,56,57], but these hormones are involved in satiation or satiety and not in hunger.

Since the HRV indices were not different between conditions at the time subjects requested their meal, it seems that hunger occurs in a similar sympathovagal state after exercise or rest.

### Postlunch results

After exercise, the glucose peak was reached more than 30 min later than after rest. Since the rate of eating was kept similar across conditions, this may indicate that gastric emptying was slowed by prior exercise. The transit time of a 3331 kJ meal with 67% fat was not previously reported to be modified by prior exercise [58]. However, mean energy intake at lunch in our study was higher (5225 ± 998 kJ), and the fat load was lower (45%) than in this previous study, so that this explanation cannot be excluded.

Based on the incremental profiles, exercise induced a higher postprandial glucose peak level and a 48% increase in total glucose response to the meal compared with the rest condition. Although our subjects were young and healthy, this could be interpreted as a detrimental effect of exercise since a sustained elevated postprandial glucose level is now considered an independent cardiovascular risk factor [59]. However, the absolute values preclude such a conclusion because the glucose AUC over the 180 min was not different between conditions. These results suggest that this response might be explained by a lower basal glucose level after the exercise session, although it failed to reach statistical significance. It has been demonstrated that exercise consisting of a sufficient workload before a meal can induce a lower postprandial insulin level [5,7,13,14]. This has been found to be partially explained by a reduced second-phase insulin secretion [9] and higher insulin clearance [60]. Therefore, the exercise-induced increase in postprandial glucose response might be the result of reduced glucose transport into the muscles due to both lower insulin and a greater glucose output from the splanchnic region, which was facilitated by prior exercise [10]. The increased post-exercise fatty oxidation observed in the muscle might also contribute to the increase in postprandial glucose. It has been documented that increasing fatty acid levels induces an increase in fat oxidation and decreased glucose oxidation via the inhibition of glucose transport/phosphorylation [61]. The involvement of fat oxidation according to the well-known Randle cycle [62] has, to this day, only been demonstrated indirectly [63]. That fat oxidation remained elevated by 46% in the exercise condition when compared to the rest condition 180 min after the meal supports this hypothesis. However, it is true that no study has yet demonstrated an increase in plasma glucose concentration due to an increase in fatty acid concentration. This absence has been attributed to a compensatory increase in insulin secretion [64], a phenomenon that might not occur following exercise.

Although increased heart rate indirectly suggests some sympathetic activation, the differences in LF/HF of the HRV analyses failed to reach significance. Interestingly, we actually observed that the previously described postprandial vagal withdrawal [28] was much more pronounced in the exercise than in the rest condition. The fact that this difference involved not only the HFms^2 ^but also the HFnu suggests its vagal origin. Pancreatic β-cells express several G protein-coupled receptors that respond to parasympathetic innervation and in turn increase glucose-stimulated insulin secretion [65]. Recently, the importance of vagal activity was demonstrated using atropine which partially blocked insulin sensitivity in the postprandial period [20]. Thus, the effect of prior exercise on postprandial metabolism could involve postprandial vagal withdrawal and sympathetic activation, both of which would result in a transiently blunted glucose tolerance mediated by reduced insulin secretion [21,66]. Importantly, this decrease in global HRV is not consecutive to a lower ability to detect variability [67]. HRV is lower after exercise than after rest because exercise induces a significant decrease in parasympathetic activity and an increase in sympathetic activity (leading to the increased HR) but with a global diminution in global HRV [68]. However, changes in vagal activity that are determined using HRV (i.e., heart branch) are not always associated with consistent changes in hormones, which are known to be highly dependent on the abdominal vagal activity [69]. Therefore, this hypothesis requires further investigation.

## Conclusion

In young and healthy male adults, a meal is requested during the same preprandial glucose decline after exercise than after rest but in a metabolic state that is characterized by higher oxidation of fat. This difference is still observed 3 h after meal consumption and is accompanied by a higher glucose response to the meal. Our results suggest that a shift in the sympathovagal balance toward a sympathetic predominance may contribute to this effect of exercise.

## List of abbreviations used

(AUC): Area under the curve; (CHO): carbohydrate; (EE): energy expenditure; (HF): high frequency; (EX): exercise; (HR): heart rate; (HRt_max_): predicted theoretical maximal heart rate; (HRV): heart rate variability; (LF): low frequency; (PPGD): preprandial glucose decline; (RT): rest; (VAS): visual analogue scale; (VO_2max_): maximal oxygen consumption; (RER): respiratory exchange ratio.

## Competing interests

The authors declare that they have no competing interest.

## Authors' contributions

KC managed the subjects, collected and analyzed the data and contributed to their interpretation and the writing of the manuscript. AP helped to collect the data, supervised the analysis and the interpretation of the HRV indices and reviewed the manuscript. DC designed the study, managed the glucose monitoring, helped to collect, analyze and interpret the data and wrote the first draft of the manuscript. All authors read and approved the final manuscript.
